# Quiet Quitting and Disengagement-Related Constructs in Nursing: A Theory-Informed Scoping Review

**DOI:** 10.3390/healthcare14142145

**Published:** 2026-07-16

**Authors:** Magdalini Soutzidou, Ioannis Kouroutzis, Theodosios Paralikas, Anna Mavroforou, Maria Malliarou

**Affiliations:** 1 Healthcare Innovation Laboratory, Department of Nursing, University of Thessaly, Larissa-Trikala Ring Road, 41500 Larissa, Greece; linasoutzidou@gmail.com (M.S.); ikouroutzis@uth.gr (I.K.); 2 Department of Nursing, University of Thessaly, Larissa-Trikala Ring Road, 41500 Larissa, Greece; paralikas@uth.gr (T.P.); amavroforou@uth.gr (A.M.)

**Keywords:** quiet quitting, nursing, work disengagement, work engagement, patient safety, quality of care, missed nursing care, scoping review

## Abstract

**Highlights:**

**What are the main findings?**

- Quiet quitting in nursing is conceptualized as reduced discretionary effort within formal role boundaries and is situated within adverse workplace conditions rather than being treated solely as an individual motivational problem; only two empirical studies have measured it directly among nurses.
- Disengagement-related constructs, including low work engagement, reduced vigor, and low-effort profiles, were associated with poorer perceived quality of care, lower nurse safety behavior, and more missed nursing care in some studies, but no study has directly linked quiet quitting to missed nursing care.

**What are the implications of the main findings?**

- Quiet quitting should be approached as a potential healthcare system and organizational signal rather than an individual motivational defect, with missed nursing care identified as the most clinically salient and least directly tested outcome domain.
- Future research requires direct measurement of quiet quitting, longitudinal and multilevel designs, and outcome data extending beyond self-reports to determine whether and how quiet quitting is associated with patient safety and care quality.

**Abstract:**

**Background/Objectives**: Quiet quitting (QQ) has become a prominent concept in healthcare workforce discourse, yet its meaning, measurement, and relevance to care delivery remain unclear. This theory-informed scoping review mapped recent evidence on QQ and related disengagement-related constructs among nurses, focusing on conceptualization, measurement, and associations with nursing performance, patient safety, quality of care, and missed nursing care. **Methods**: The review was guided by the Population–Concept–Context (PCC) framework and reported in accordance with the Preferred Reporting Items for Systematic Reviews and Meta-Analyses Extension for Scoping Reviews (PRISMA-ScR). Web of Science, Scopus, PubMed, the Cochrane Library, and CINAHL were searched on 19 March 2026 for empirical, peer-reviewed, English-language studies involving nurses and published from 2022 onward. Eligible studies examined QQ or adjacent disengagement-related constructs in relation to nursing performance or care-delivery outcomes. Data were charted narratively and appraised using the Mixed Methods Appraisal Tool (MMAT). **Results**: Seven studies met the inclusion criteria; two examined QQ directly. Direct evidence conceptualized QQ as reduced discretionary effort within formal role boundaries and situated it within adverse workplace conditions. Adjacent evidence addressed low work engagement, reduced vigor, low-effort and engagement–burnout profiles, which in some studies were associated with poorer perceived quality of care, lower safety behavior, poorer performance, or more missed care. No included study directly examined QQ in relation to missed nursing care. **Conclusions**: QQ in nursing should be reframed from a workforce label into a care-delivery question. Establishing its clinical relevance will require direct testing of whether QQ is associated with missed nursing care, a link that has not yet been examined directly.

## 1. Introduction

Healthcare systems are expected to deliver safe, high-quality care amid scarce resources, rising patient complexity, and persistent workforce shortages. Nurses absorb much of this pressure directly: as the clinicians closest to patients, their vigilance supports timely detection of deterioration, continuity of care, and everyday patient safety.

In many settings, this work takes place under demanding conditions, including heavy workload, staffing gaps, workplace violence, fatigue, limited recovery time, and repeated exposure to trauma [1,2,3,4]. These conditions not only affect performance and retention but also the extent to which nurses remain psychologically and motivationally invested in their work. When leaving is not viable or desired, withdrawal may become less visible: reduced emotional investment, diminished initiative, or effort confined strictly to formal duties. This is clinically relevant because related workforce and organizational strains have been associated with missed nursing care, poorer perceived quality of care, and adverse patient outcomes [5,6,7,8].

QQ represents one specific form of within-role disengagement. In nursing, it has been described through minimal compliance with job expectations, reduced discretionary effort, and psychological or emotional detachment from work [9,10,11]. The recent literature situates QQ within organizational and psychological pressures, including excessive demands, poor leadership, perceived unfairness, and misalignment between personal and organizational expectations [12,13,14,15]. In this sense, QQ is not simply a new label for low motivation; it describes reduced work investment while employment and formal role occupancy continue [11,16]. It differs from burnout, which is typically defined by emotional exhaustion, depersonalization or cynicism, and reduced professional efficacy, and from low work engagement, which centers on diminished vigor, dedication, and absorption. QQ instead refers more specifically to the selective scaling back of discretionary effort while formal duties continue to be met [17,18].

In nursing, however, formal task completion cannot be equated with safe care. QQ is unlikely to appear as an outright refusal of essential patient care; it is more likely to emerge in the discretionary, relational, and anticipatory parts of practice, including initiative, peer support, care coordination, and participation in quality-improvement work [19]. These activities often remain less visible than task completion, yet they sustain continuity, teamwork, and safety. They also align closely with nursing-specific process outcomes such as missed nursing care and care left undone, which were developed to capture omissions, delays, and rationing in care delivery. This is why the present review focuses on nursing rather than adopting a broad scope for healthcare professionals: the relevant process outcomes have been developed and validated specifically for nursing care, whereas medical practice is organized through different role structures and outcome measures [20,21].

Clear conceptual boundaries are therefore necessary. QQ differs from turnover intention because it does not primarily concern an intention to leave the job [15,22,23]. It may overlap empirically with reduced motivation, disengagement, or withdrawal, but it remains conceptually distinct from each of these constructs.

For this reason, the present review treats QQ and adjacent disengagement-related constructs as related but non-equivalent phenomena. Kahn’s theory of personal disengagement provides the conceptual anchor, describing how individuals may remain in a role while withdrawing physically, cognitively, or emotionally from their work [24,25]. Constructs such as low engagement, reduced vigor, and psychological withdrawal were therefore considered relevant only when they shared this work-role logic and were linked to nursing performance, patient safety, quality of care, or missed nursing care.

The Job Demands–Resources (JD-R) model and Conservation of Resources theory further suggest that nurses may reduce discretionary effort to protect emotional, cognitive, and psychological resources when work demands exceed available resources [1,13,26,27,28,29,30]. Social exchange, psychological contract, and self-determination perspectives add that fairness, reciprocity, autonomy, competence, and relatedness may shape this process [25,31,32,33,34,35]. These frameworks are used here to clarify construct boundaries and guide interpretation, not to define eligibility criteria.

Conceptual imprecision also creates a measurement problem. The quiet quitting scale (QQS) developed by Galanis and colleagues operationalizes QQ through detachment, lack of initiative, and motivation [9], whereas other instruments emphasize minimum effort, avoidance of unpaid extra work, behavioral restriction, or a deliberate decision not to exceed core responsibilities [23,31,36]. Adjacent constructs follow different measurement traditions: work engagement is typically measured through vigor, dedication, and absorption; burnout through exhaustion, depersonalization, and reduced accomplishment; and missed nursing care through instruments such as MISSCARE, BERNCA, and care-left-undone items [5,6,8,17]. Studies may therefore appear to address the same phenomenon while actually measuring different forms or levels of work investment.

Against this backdrop, the nursing evidence base remains recent, sparse, and fragmented. Broader organizational reviews already note that QQ lacks a settled conceptual framework and that its boundaries with disengagement and burnout are still debated [15,22,37]. Within nursing specifically, direct studies remain few, and the available evidence is dispersed across conceptual, antecedent-focused, workforce-focused, and adjacent disengagement-related studies [29,30,33,38,39].

Several adjacent efforts have begun to address this terrain. A combined concept analysis and scoping review protocol on QQ among hospital-based healthcare professionals has been registered [40], and a recent integrative review synthesized nursing evidence on QQ’s antecedents, consequences, and workforce outcomes, including turnover intention and organizational impact [41]. A cross-profession meta-analysis examined QQ antecedents across industries and identified workplace support, organizational injustice, and burnout as key predictors, although only a subset of included studies involved nurses or other healthcare workers, and the focus remained on antecedents rather than care-delivery outcomes [26]. Two concept analyses have examined QQ in nursing directly, clarifying its defining attributes and distinguishing it from burnout and turnover, but neither used a scoping review methodology with predefined eligibility criteria focused on care-delivery outcomes [10,11].

Taken together, these works show that QQ in nursing has begun to receive conceptual and workforce-focused attention. What remains unresolved is a more specific care-delivery question: Have direct QQ and adjacent disengagement-related evidence been mapped against nursing performance, patient safety, quality of care, and, especially, missed nursing care, care left undone, and omitted care? This gap matters because nursing care depends on discretionary, relational, anticipatory, coordinating, and safety-sensitive work that extends beyond minimum task completion. Reduced initiative, emotional detachment, and passive role performance may therefore become visible in care-delivery processes before they appear in distal patient outcomes.

This review responds to that gap by treating QQ as a nursing care-delivery question rather than a workforce label. This framing does not assume that QQ causes poor or missed care. Instead, it asks whether the construct has been examined against outcomes that reflect how nursing care is delivered. Direct QQ evidence is therefore distinguished throughout from adjacent disengagement-related evidence, with the latter used to identify where empirical gaps remain. The present review differs from the closest available prior syntheses by applying a PRISMA-ScR-guided, care-delivery-focused mapping of direct QQ evidence and adjacent disengagement-related evidence in nursing, with missed nursing care treated as a predefined outcome domain.

Accordingly, this theory-informed scoping review maps how QQ and closely related disengagement-related constructs are conceptualized, operationalized, and measured among nurses, and how they are associated with nursing performance, patient safety, quality of care, and missed nursing care. It addresses four questions: (1) How are QQ and closely related disengagement-related constructs conceptualized in nursing research? (2) How are QQ and disengagement-related constructs operationalized and measured among nurses? (3) What associations are reported between QQ or disengagement-related constructs and nursing performance, safety behavior, patient safety, or quality-of-care outcomes? (4) What associations are reported between QQ or disengagement-related constructs and missed nursing care, including care left undone or omitted care?

## 2. Materials and Methods

This review used a theory-informed scoping review design. A scoping approach was selected because QQ in nursing is emerging, conceptually heterogeneous, and supported by limited direct evidence, alongside a broader body of studies on adjacent disengagement-related constructs. Scoping reviews are appropriate for mapping the extent, range, and nature of evidence, clarifying concepts, examining how research has been conducted, and identifying knowledge gaps, rather than estimating intervention effects or producing pooled findings [42,43,44].

This design was more appropriate than an effectiveness-oriented systematic review because the review did not evaluate a specific intervention. It was also more appropriate than a formal concept analysis because the aim was not to produce a definitive definition of QQ, but to examine how QQ and related constructs have been used, measured, and linked to care-delivery outcomes in recent empirical nursing research.

The review was informed by the methodological framework of Arksey and O’Malley [42] and the refinements proposed by Levac et al. [43]. The reporting was guided by PRISMA-ScR [45]. A completed PRISMA-ScR checklist is provided as Supplementary Table S1.

No review protocol was prospectively registered or published, which is acknowledged as a limitation. However, a predefined methodological plan was established prior to full-text eligibility assessment and synthesis, and methodological decisions were documented throughout the review process to support transparency and reproducibility.

### 2.1. Eligibility Criteria

Eligibility criteria were structured using the PCC framework, which is recommended for scoping reviews that aim to define the population, concept, and context of interest [46]. The population comprised registered nurses and licensed nursing staff involved in direct patient care in clinical healthcare settings. The concept comprised QQ and constructs closely related to disengagement. The context comprised healthcare-delivery organizations, including hospitals, critical care units, primary care services, community care, and long-term care facilities.

The theory-informed character of the review did not function as an additional eligibility criterion. Instead, it informed the interpretation of construct boundaries, particularly the distinction between direct QQ evidence and evidence on adjacent disengagement-related constructs. Theories were used heuristically to interpret construct boundaries and explanatory pathways rather than as formal deductive analytic frameworks.

Because QQ remains inconsistently used in the peer-reviewed nursing literature, eligibility followed a two-route logic. First, studies directly examining QQ among nurses were eligible when they contributed evidence relevant to the review questions, particularly by clarifying conceptualization, operationalization, or measurement of QQ in nursing, or by reporting associations with nursing performance, safety behavior, patient safety, quality of care, missed nursing care, care left undone, or omitted care. Direct QQ studies focused solely on antecedents, general psychosocial correlates, organizational correlates, or workforce outcomes were not included as primary evidence unless they contributed substantively to this conceptual, measurement, or care-delivery focus. Where relevant, such studies were used only for background or contextual discussion.

Second, studies examining adjacent disengagement-related constructs were only eligible when the construct was conceptually consistent with disengagement-like manifestations and empirically linked to at least one relevant care-delivery outcome. This broader route was necessary because direct QQ evidence in nursing remains limited and conceptually emergent; restricting eligibility to studies explicitly labeled as QQ would have produced too narrow an evidence base to evaluate potential care-delivery relevance. Adjacent constructs were therefore included to map theoretically and clinically related manifestations, while still preserving the distinction from direct QQ evidence. Eligible constructs included low or diminished work engagement, reduced vigor, psychological or emotional withdrawal, diminished initiative, minimal compliance, reduced discretionary effort, and burnout–engagement profiles. Burnout itself was not treated as equivalent to QQ; it was eligible only when explicitly connected to disengagement-like manifestations or burnout–engagement profiles and linked to a relevant outcome.

Eligible studies were empirical, quantitative, qualitative, or mixed-methods studies, published in English-language peer-reviewed journals from 2022 onward and available in full text. Reviews were not included as primary evidence, although relevant reviews were screened for reference mining. Full eligibility criteria are summarized in Table 1.

### 2.2. Information Sources and Search Strategy

A structured literature search was conducted in Web of Science, Scopus, PubMed, the Cochrane Library, and CINAHL Plus with full text. Web of Science and Scopus were included for broad multidisciplinary coverage, but they do not provide the same depth of nursing-specific indexing as CINAHL. PubMed was included as the core biomedical database, and the Cochrane Library was searched to capture relevant systematic reviews and methodological sources. This combination is also consistent with database selection in comparable reviews on this topic [40,41]. A supplementary Google Scholar search was conducted to increase sensitivity, and backward reference checking of relevant studies and reviews was undertaken as an additional method. The final search was conducted on 19 March 2026.

The search strategy reflected the two-route eligibility logic. Search terms covered four concept blocks: quiet quitting and disengagement-related constructs; nursing population; nursing performance, patient safety, and quality-of-care outcomes; and missed nursing care, care left undone, omitted care, or rationed care. Free-text terms were adapted to each database, and controlled vocabulary was used where available, particularly in PubMed and CINAHL. Boolean operators, truncation, phrase searching, and database-specific subject headings were applied as appropriate. Full database-specific search strategies are provided in Supplementary Table S2.

The search was restricted to studies published from 2022 onward because the term QQ entered widespread public and, subsequently, peer-reviewed workforce and nursing discourse following its viral emergence that year, within the broader post-pandemic period of workforce disruption [40]. The earlier literature on work engagement, burnout, staffing, missed nursing care, patient safety, and quality of care was only used for conceptual and contextual positioning, unless it met the review’s timeframe and eligibility criteria. The same timeframe was applied across both eligibility routes rather than extending it selectively for adjacent constructs, which would have created two evidence bases assessed under different temporal boundaries and complicated synthesis. This choice means that established, long-standing evidence on work engagement and burnout predating 2022 was not systematically captured, representing a trade-off between historical depth and methodological consistency across the two eligibility routes.

Where available, filters were applied for English language, peer-reviewed journal articles, and a publication date from 2022 onward. Full-text availability was not used as an initial search filter to avoid reducing search sensitivity during identification. When database filters were unavailable or inconsistent, the same restrictions were applied during screening and the full-text eligibility assessment.

Google Scholar searches used simplified combinations of terms related to QQ, work engagement, disengagement, nurses, patient safety, quality of care, performance, missed nursing care, care left undone, and omitted care. Retrieved records were screened by the title and a snippet, and potentially relevant full texts were assessed using the same eligibility criteria as database records. Because Google Scholar does not support the same level of reproducibility as bibliographic databases, this supplementary search was used to increase sensitivity and was documented separately from database searching. The search date, simplified search strings, and screening range for the Google Scholar search are reported in the Supplementary Materials.

### 2.3. Study Selection

Study selection proceeded in two stages: initial screening of titles and abstracts, followed by full-text assessment. Records that clearly did not meet the eligibility criteria were excluded at the first stage, while uncertain records were retained for full-text review. At full-text assessment, each excluded report was assigned one primary reason for exclusion. For studies examining adjacent disengagement-related constructs, eligibility required both an explicit disengagement-like manifestation, such as reduced engagement, diminished initiative, or psychological withdrawal, and an empirical link to at least one relevant care-delivery outcome; the absence of either element resulted in exclusion. Screening was conducted by two reviewers, with uncertainties, including borderline judgments on whether a construct was sufficiently aligned with disengagement-like manifestations, resolved through discussion and consensus.

From database searches and the initial supplementary Google Scholar search, 3261 records were identified. Of these, 2912 were retrieved from bibliographic databases: Web of Science (*n* = 130), Scopus (*n* = 144), PubMed (*n* = 1754), the Cochrane Library (*n* = 1), and CINAHL (*n* = 883). A further 349 records were identified through Google Scholar. After the removal of 540 duplicates, 2721 records remained. Nine records were removed before screening because they represented clearly ineligible record types or indexing artifacts. The remaining 2712 records were screened by title and abstract; 2642 were excluded, and 70 reports were sought for retrieval. All 70 reports were retrieved and assessed for full-text eligibility.

At full-text assessment, 64 reports from the database and supplementary search pathway were excluded for the following primary reasons: study design (*n* = 8), language (*n* = 1), population (*n* = 5), concept (*n* = 36), and outcomes (*n* = 14). Six studies from this pathway were included.

Backward reference checking was reported as a separate supplementary pathway. It identified 15 additional records, all of which were retrieved and assessed. Fourteen were excluded because they did not report outcomes relevant to nursing performance, safety behavior, patient safety, quality of care, missed nursing care, care left undone, or omitted care. One additional study was included through this process.

Overall, seven studies were included in the final review corpus: six from database and supplementary searching and one from backward reference checking. The small corpus itself is an evidence-mapping finding. The search strategy was deliberately broad and covered both direct QQ terms and adjacent disengagement-related constructs, yet most potentially relevant records did not report associations with nursing performance, patient safety, quality of care, missed nursing care, care left undone, or omitted care. Backward reference checking showed the same pattern: fourteen of fifteen additional records were excluded because they lacked a relevant outcome association. This supports the interpretation that the corpus reflects the emerging and outcome-specific nature of the literature, not simply a narrow search strategy.

The corpus size is also consistent with related syntheses on this topic. An integrative review identified nine studies [41] and a concept analysis using a less restrictive interpretive approach identified sixteen studies [11]. Reviews reporting larger corpora have generally used broader eligibility criteria, included antecedents or workforce outcomes, or adopted concept-analytic rather than care-delivery-focused design [10].

**Figure 1:**
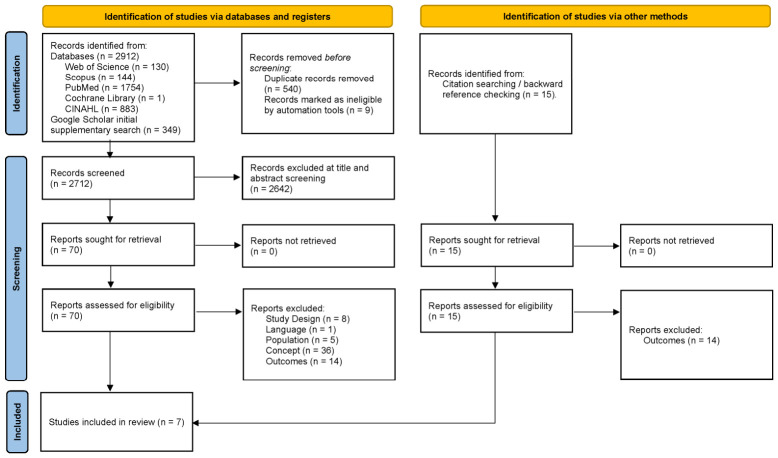
Flow diagram of the study selection process, adapted from the PRISMA 2020 flow diagram and reported in accordance with PRISMA-ScR [45,47].

The full study selection process is presented in Figure 1.

### 2.4. Data Charting Process and Data Items

Data charting was conducted for all included studies using a standardized form developed around the PCC framework, eligibility criteria, and review questions. The form was refined iteratively as familiarity with the studies increased. Charting was conducted by one reviewer and independently verified by a second reviewer. Discrepancies or uncertainties were resolved through re-examination of the full text.

Charted items included bibliographic information, study aim, country, study design, sample characteristics, clinical setting, construct examined, measurement instrument, outcome domain, and whether the study addressed QQ directly or through an adjacent disengagement-related construct. For direct QQ studies, charting focused on definition, perception, measurement, operationalization, reported levels, prevalence, and relevant correlates. For adjacent construct studies, charting focused on conceptual relevance to disengagement-like manifestations, outcome assessments, and direction of associations.

Because the included studies were heterogeneous, findings were charted narratively rather than as standardized numerical effect estimates. Quantitative associations were summarized by direction and relevance to the review questions. Qualitative findings were charted as themes or interpretations relevant to QQ, disengagement-related manifestations, nursing performance, care delivery, or patient safety.

### 2.5. Synthesis of Results

The synthesis was descriptive and narrative, supported by a theory-informed interpretive approach. First, studies were mapped by PCC, measurement approach, and outcome domain. Second, findings were grouped into categories aligned with the review questions.

No meta-analysis was conducted because of heterogeneity in the study design, construct terminology, measurement instruments, and outcomes. Reported statistical associations were used only to map the direction of association and were not interpreted causally. Qualitative findings were summarized narratively.

The theory-informed component functioned as an interpretive lens rather than an additional eligibility framework. It helped clarify construct boundaries, distinguish direct QQ evidence from adjacent construct evidence, and identify where empirical links with nursing performance and care-delivery outcomes were present, absent, or insufficiently specified.

### 2.6. Methodological Quality Appraisal

Methodological quality appraisal was undertaken to contextualize the robustness and limitations of the included evidence. Although critical appraisal is optional in scoping reviews, PRISMA-ScR recommends reporting the rationale, methods, and use of appraisal findings when appraisal is conducted [45]. Appraisal was not used as an eligibility criterion, and no study was excluded on the basis of methodological quality [44].

Included studies were appraised using the MMAT [48], which supports appraisal across qualitative, quantitative, and mixed-methods designs. This was appropriate because the corpus included quantitative observational and cross-sectional studies, one longitudinal study, and one exploratory sequential mixed-methods study. Appraisal was conducted by one reviewer. Uncertain ratings were checked against the full text and MMAT guidance before final coding.

Across the included studies, stronger features included clear research aims, appropriate alignment between objectives and methods, established or clearly described measurement instruments, and suitable analytical procedures. The main limitations included the predominance of cross-sectional designs, reliance on self-reported outcomes, non-probability or context-specific sampling, limited information on nonresponse bias, and heterogeneity in the conceptualization and operationalization of QQ and adjacent constructs. The longitudinal design of Gillet et al. [49] strengthened the evidence base, although attrition and self-report measurement remained relevant considerations.

Overall, all studies were considered methodologically acceptable for evidence mapping. The appraisal findings reinforced the need for cautious, non-causal interpretation of associations between quiet quitting, disengagement-related constructs, nursing performance, patient safety, quality of care, and missed nursing care outcomes.

These methodological features carry specific implications for confidence in the review findings. The predominance of cross-sectional designs means that reported associations reflect concurrent relationships rather than temporal sequence, so findings cannot support inferences about whether disengagement-related manifestations precede or follow changes in performance, safety behavior, or care delivery. Reliance on self-reported outcomes introduces the possibility of common method bias and social desirability effects, which may inflate or obscure associations between disengagement-related constructs and care-related outcomes. Non-probability and context-specific sampling further limit confidence in the generalizability of findings across nursing populations, healthcare systems, and cultural contexts. Taken together, these features indicate that the associations reported in this review should be interpreted as indicative signals within a still-developing evidence base rather than as robust or generalizable effects. Appraisal results are presented in Table 2.

## 3. Results

### 3.1. Characteristics of the Included Studies

The final corpus comprised seven studies conducted in six countries: the Philippines, Canada, China, Greece, Israel, and the United States (Table 3). Five studies used cross-sectional designs, including descriptive, correlational, nested, and online survey approaches [51,52,53,54,55]. One study used an exploratory sequential mixed-methods design combining a qualitative case-study phase with a descriptive cross-sectional survey [50]. One study used a longitudinal design with two measurement points 12 months apart [49]. Sample sizes ranged from five nurses in the qualitative phase of Abdullah and Bangcola [50] to 957 oncology nurses in Ma et al. [55]. All studies were conducted in hospital-based or clinical nursing contexts.

Two studies examined QQ directly: Abdullah and Bangcola [50] focused on QQ among millennial and Generation Z nurses, and Moisoglou et al. [53] examined QQ alongside workplace gaslighting. The remaining five studies examined adjacent constructs, including work engagement, low-effort engagement profiles, nurse vigor and exhaustion, and combined engagement–burnout profiles [49,51,52,54,55]. This distribution is an important evidence-mapping finding: QQ has begun to be studied directly in nursing, but not yet as a care-delivery construct. At present, adjacent evidence provides most of the empirical basis for considering its potential clinical relevance. Study characteristics are summarized in Table 3.

### 3.2. Conceptualization, Operationalization, and Measurement of QQ and Adjacent Disengagement-Related Constructs

Across the included studies, QQ and its adjacent constructs were conceptualized, operationalized, and measured through two evidence routes: direct QQ evidence and adjacent evidence (Table 4).

Direct QQ evidence came from Abdullah and Bangcola [50] and Moisoglou et al. [53]. Both studies framed QQ as a disengagement-related work behavior in which nurses remain in role, meet formal job requirements, and limit effort beyond those requirements, while situating QQ within workplace conditions rather than treating it as a purely individual attitude. Abdullah and Bangcola [50] described QQ as meeting job requirements without striving for overachievement. Moisoglou et al. [53] conceptualized it as deliberately scaling back discretionary effort and limiting performance to the formal job description.

The remaining five studies contributed adjacent evidence. Work engagement was conceptualized as a positive work-related state involving vigor, dedication, and absorption [51,52,55]. Srulovici and Yanovich [54] distinguished vigor from exhaustion within a JD-R pathway, while Gillet et al. [49] examined combined profiles of engagement and burnout among novice nurses. Across all seven studies, QQ itself was consistently defined as reduced discretionary effort within formal role boundaries; low engagement, reduced vigor, low dedication, low-effort profiles, and combined engagement–burnout profiles were treated as related but non-equivalent constructs.

Operationalization and measurement varied across the included studies (Table 4). Quiet quitting was measured directly in two studies, but with different approaches. Abdullah and Bangcola [50] used an exploratory mixed-methods procedure in which qualitative interviews informed a subsequent survey, with QQ level classified through a weighted scoring system, while Moisoglou et al. [53] measured quiet quitting using the 9-item QQS [9]. The remaining five studies operationalized adjacent constructs through work engagement scales, vigor and exhaustion measures, or person-centered engagement and burnout profiles. These measures provide inverse or proximal evidence on disengagement-related functioning, not direct measurement of QQ. Direct measurement of QQ in nursing, therefore, remains limited, and findings from adjacent constructs should be read as conceptually informative rather than equivalent evidence.

### 3.3. Associations with Nursing Performance, Safety Behavior, Patient Safety, and Quality of Care

Five studies reported outcomes related to perceived quality of care, perceived patient safety, nurse safety behavior, patient adverse events, and in-role and extra-role performance (Table 5). Evidence in this domain was mostly adjacent; no included study tested QQ itself as a predictor of nursing or care-delivery outcomes.

Moisoglou et al. [53] provided the only QQ-related evidence in this domain, though indirectly: QQ itself was not tested as a predictor of perceived quality of care or patient safety. Instead, workplace gaslighting, an exposure associated with higher QQ, was also associated with lower odds of good-to-excellent perceived quality of care and perceived patient safety.

Among the work engagement studies, Wei et al. [51] found that nurses reporting high perceived quality of care had higher total and dimensional engagement scores than those reporting low perceived quality of care, meaning lower engagement corresponded to poorer perceived quality of care. Falguera et al. [52] found a more specific pattern: dedication was the only engagement dimension significantly associated with perceived quality of care, while vigor, absorption, and overall engagement were not, and no engagement dimension was significantly associated with patient adverse events.

Ma et al. [55] linked engagement profiles to nurse safety behavior: the low effort and coping type showed the lowest safety behavior scores, with the moderately balanced and highly efficient and focused types showing progressively higher levels. Gillet et al. [49] linked combined engagement–burnout profiles to nursing performance, finding that the profile characterized by low dedication, reduced efficacy and high cynicism was associated with poorer in-role and extra-role performance than more adaptive profiles.

Overall, several disengagement-related manifestations were associated with poorer care-delivery or performance outcomes, although the pattern varied by construct and outcome. Lower dedication was associated with poorer perceived quality of care, low-effort engagement profiles with lower safety behavior, and disengagement-prone engagement–burnout profiles with poorer performance. These findings provide adjacent evidence only. No included study directly tested QQ as a predictor of nursing performance, patient safety, or quality-of-care outcomes.

### 3.4. Associations with Missed Nursing Care, Care Left Undone, and Omitted Care

Two studies examined missed nursing care, care left undone, or omitted care in relation to adjacent disengagement-related constructs (Table 6). More specifically, Falguera et al. [52] examined missed nursing care as care left undone during the last shift because of a lack of time, using the 13-item instrument developed by Lake et al. [21] and found no significant association between vigor, dedication, absorption, or overall engagement and missed nursing care.

Srulovici and Yanovich [54] examined missed nursing care using the 22-item MISSCARE survey [20] within a JD-R framework: lower vigor was associated with more frequent missed nursing care, supporting the motivational pathway, while exhaustion was not associated with missed care in the final model, leaving the energetic pathway unsupported.

Evidence in this domain was therefore limited and mixed. One study linked lower vigor to more frequent missed care, whereas the other found no significant association between engagement dimensions and care left undone. No included study directly examined the relationship between QQ and missed nursing care.

## 4. Discussion

This theory-informed scoping review examined QQ among nurses as an emerging healthcare quality, patient-safety, and care-delivery question, rather than only as a workforce or organizational behavior construct. By distinguishing direct QQ evidence from adjacent evidence, the review mapped how QQ and related constructs have been conceptualized, measured, and linked to nursing performance, patient safety, quality of care, and missed nursing care. The discussion below interprets the main findings, considers their conceptual and measurement implications, and outlines priorities for future nursing research.

### 4.1. Discussion of Findings

This review addressed four questions concerning the conceptualization, measurement, and care-delivery relevance of QQ and adjacent disengagement-related constructs in nursing. Direct evidence characterized QQ as reduced discretionary effort within continued role occupancy, situated within adverse workplace conditions rather than individual motivation alone (RQ1). Only two of the seven included studies that measured QQ directly, using either a locally developed scoring approach or a standardized scale (RQ2) [50,53]. The main finding is therefore not that QQ has established effects on nursing care, but that its direct relationship with nursing performance, patient safety, quality of care (RQ3), and missed nursing care (RQ4) remains largely untested.

This gap is consistent with recent nursing discussions describing QQ as conceptually and empirically unsettled, particularly regarding its boundaries, antecedents, measurement, and consequences [10,11,19]. This review extends the literature by showing that QQ has begun to be defined and measured among nurses, though most available evidence still concentrates on antecedents, correlates, and workforce outcomes rather than care delivery. This pattern underscores the boundary of the present evidence base rather than establishing a developmental trajectory for the construct. The present review positions QQ at exactly this transition point.

A clear asymmetry emerged between direct and adjacent evidence. The two direct QQ studies mainly clarified how the construct is perceived, measured, and situated within adverse workplace conditions. The five adjacent studies showed that lower work engagement, reduced dedication, low-effort engagement profiles, and disengagement-prone engagement–burnout profiles were associated in some cases with poorer perceived quality of care, lower nurse safety behavior, poorer in-role and extra-role performance, or increasingly missed nursing care [49,51,52,53,55]. These constructs map QQ’s empirical neighborhood, but they cannot substitute for direct measurement. Studies that measure QQ directly are needed to determine whether it has distinct associations with care-delivery outcomes.

This distinction has direct interpretive consequences for the findings reported above. Associations observed for low engagement or burnout-related profiles cannot simply be assumed to hold for QQ itself, since these constructs capture different underlying phenomena. Maintaining this boundary, therefore, sharpens rather than weakens the conclusions of this review by making explicit which findings describe QQ directly and which describe conceptually adjacent but distinct phenomena.

Missed nursing care emerged as the most important untested outcome domain. Prior nursing research has established missed care, care left undone, and rationed care as process-level indicators through which staffing constraints, time pressure, and poor practice environments become visible as omissions or delays in necessary care [5,6,8,21], yet no included study examined QQ in relation to any of these outcomes directly. The strongest conclusion is therefore not that QQ could shape missed care, but that missed care is the outcome domain where QQ’s clinical relevance is most directly testable.

### 4.2. QQ as Work Recalibration Within Strained Nursing Work Systems

In nursing, QQ can be interpreted as a form of work recalibration within strained work systems. In the direct QQ studies, nurses remained in their roles while limiting discretionary effort, avoiding overachievement, and restricting work investment to formal job requirements [50,53]. This does not imply abandonment of core professional responsibilities. Rather, QQ reflects selective narrowing of effort, initiative, emotional investment, and willingness to go beyond formal expectations.

This distinction matters because healthcare organizations rely on forms of nursing work that are partly discretionary but operationally essential: supporting colleagues, mentoring less experienced staff, communicating proactively, participating in quality improvement, and sustaining relational care [10,11,19]. QQ is therefore relevant not because it necessarily involves refusal of essential care, but because it concerns the space between minimum task completion and fully engaged professional practice.

This interpretation reduces the risk of individualizing or moralizing disengagement. The direct QQ studies situated the phenomenon within adverse workplace conditions [50,53] and wider evidence similarly links QQ with unfavorable work environments and strained organizational conditions [19,27,29,33,39]. QQ is therefore better understood as a potential signal of work-system strain than as an individual motivational defect.

Established theory supports this interpretation. The JD-R model suggests that sustained demands, such as heavy workload, understaffing, and time pressure, along with organizational change, may reduce engagement and contribute to reduced work investment when adequate resources are lacking [1,2,28]. Kahn’s framework [24] further suggests that adverse work conditions can reduce psychological meaningfulness, safety, and availability, making it more likely that individuals remain physically present while withdrawing emotionally, cognitively, or motivationally from the work role, a process Kahn describes as personal disengagement. In this hierarchy, withdrawal describes the underlying process; disengagement describes the resulting psychological state; and quiet quitting is a one specific, nursing-relevant expression of within-role disengagement, shaped by work demands, depleted resources, perceived reciprocity, and psychological availability.

This work-system interpretation is especially important because nursing care is interdependent and safety-sensitive. Nurses monitor deterioration, coordinate care, communicate changes in patient status, support colleagues, anticipate risks, and provide relational and emotional care. Any potential relevance of QQ may therefore extend beyond the individual nurse, since reduced discretionary effort, initiative, or psychological availability can affect teamwork, communication, responsiveness, and care coordination. This does not mean QQ directly causes poor care; it explains why disengagement-related manifestations may matter differently in nursing than in less interdependent occupational settings.

**Figure 2:**
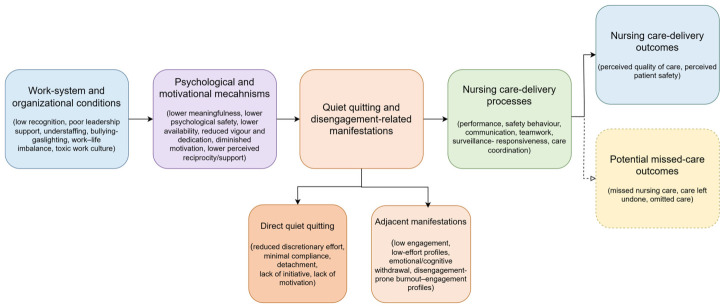
Preliminary theory-informed conceptual model of quiet quitting and disengagement-related pathways in nursing care delivery. The model links strained nursing work-system conditions with psychological and motivational mechanisms, quiet quitting and adjacent disengagement-related manifestations, care-delivery processes, and care-delivery outcomes. Solid arrows indicate theory-informed conceptual linkages, not empirically confirmed causal effects. The dashed pathway to missed nursing care, care left undone, omitted care, and rationed care indicates a theoretically plausible but not yet directly tested relationship. Box colors are used for visual differentiation between conceptual domains in the model; the domain represented by each box is indicated by its label rather than by color alone.

Based on these considerations, Figure 2 presents a preliminary theory-informed model linking strained nursing work-system conditions with psychological and motivational mechanisms, QQ and adjacent manifestations, care-delivery processes, and care-delivery outcomes. The model is conceptual, exploratory, and non-causal, and should be understood as largely theory-driven rather than evidence-driven, since no included study directly tested a pathway linking QQ to care-delivery outcomes. It organizes hypothesized relationships for future empirical testing rather than summarizing established findings; several of its proposed pathways, particularly those linking QQ to missed nursing care, have not yet been directly tested in nursing samples and should be treated as working hypotheses rather than confirmed mechanisms. This exploratory status also reflects the size of the underlying evidence base. With only seven included studies distributed unevenly across outcome domains, the model should be read as an organizing synthesis of a small and heterogeneous literature rather than as a structure with established empirical robustness.

### 4.3. Conceptual and Measurement Implications

The findings highlight the need for greater conceptual and measurement precision. Only two included studies measured QQ directly, and they used different approaches: one locally developed mixed-methods scoring procedure and one standardized QQS [50,53]. Current nursing evidence remains insufficient to determine whether QQ functions consistently across settings, countries, generations, and clinical contexts.

These limitations reflect broader challenges inherent to operationalizing and validating a recently emerged construct. Unlike work engagement or burnout, which have been measured with instruments developed and refined over several decades, QQ instruments have only recently been introduced and have not yet undergone extensive cross-cultural adaptation, longitudinal validation, or testing across diverse nursing populations [15,22,37]. Existing QQ measures have not yet accumulated extensive independent psychometric replication in diverse nursing samples, and available applications remain too limited to support strong cross-setting comparisons [50]. Until such validation evidence accumulates, comparisons of QQ levels or prevalence across studies, settings, or countries should be interpreted with caution.

A central measurement issue is whether QQ is measured directly or inferred from adjacent constructs. Work engagement measures, such as the UWES, capture vigor, dedication, and absorption; lower scores may indicate reduced energy or involvement, but they do not necessarily measure intentional restriction of discretionary effort [51,52]. Similarly, reduced vigor, low dedication, exhaustion, cynicism, and burnout–engagement profiles may reflect disengagement-related functioning, but they do not fully capture the defining boundary of QQ: continued role occupancy combined with selective withdrawal of effort beyond formal requirements. Future studies should therefore avoid using low engagement, burnout, or reduced vigor as substitutes for QQ unless the conceptual link is explicitly justified.

The dimensional structure of QQ also requires further validation. Existing nursing work has treated detachment, lack of initiative, and lack of motivation as core dimensions of the construct [9,53]. Future validation studies should clarify whether these dimensions operate as reflective indicators of an underlying latent construct or as formative components that together constitute QQ, since different dimensional models lead to different interpretations of scale scores and cross-study comparisons.

Person-centered approaches may complement direct QQ measurement by identifying patterns that total scores obscure [49,55]. Such approaches may help distinguish nurses who are broadly disengaged from those who selectively limit extra-role effort while maintaining core patient care responsibilities.

Without conceptual and psychometric precision, QQ risks becoming an umbrella label for multiple forms of distress, disengagement, and work withdrawal. A key unresolved issue is whether QQ represents a substantively distinct construct or a contemporary reframing of previously described forms of disengagement, withdrawal, or boundary setting. Although the recent nursing and organizational literature increasingly treats QQ as distinct, its discriminant validity relative to burnout, low work engagement, and other withdrawal-related constructs remains insufficiently established. Future nursing research should therefore specify whether QQ is measured directly, inferred from low engagement, or examined through adjacent disengagement-related profiles.

Table 7 summarizes core conceptual distinctions between QQ and the three adjacent constructs most frequently discussed in relation to it in this review, namely burnout, work engagement, and turnover intention. These distinctions are intended to clarify construct boundaries for the purposes of this review and should not be read as implying that the constructs are empirically independent in all nursing contexts.

### 4.4. Implications for Clinical Performance, Quality of Care, and Patient Safety

The mapped evidence suggests that disengagement-related work investment may be relevant to nursing performance, quality of care, and patient safety, although the strength and directness of evidence vary by construct and outcome. Clinical performance in nursing is not limited to completion of assigned tasks; it also involves communication, coordination, clinical judgment, accountability, responsiveness, and the relational work required to sustain safe care [56,57]. From this perspective, reduced initiative, detachment, low dedication, diminished vigor, and minimal compliance matter because they concern less visible components of nursing work that support continuity, teamwork, and safety.

The direct QQ evidence remains limited. Moisoglou et al. [53] identified workplace gaslighting as an adverse exposure associated with higher QQ and with lower odds of good-to-excellent perceived quality of care and patient safety, although QQ itself was not tested as a direct predictor of these outcomes. Adjacent evidence provides a stronger but still indirect signal: lower work engagement and dedication were associated with poorer perceived quality of care, low-effort engagement profiles with poorer nurse safety behavior, and disengagement-prone engagement–burnout profiles with poorer in-role and extra-role performance [49,51,52,55]. These findings are consistent with wider nursing research linking work engagement, burnout, workload, staffing, leadership, and practice environments with performance, quality of care, patient-safety indicators, and patient-related outcomes [58,59], although results were not uniform; dedication, for instance, predicted perceived quality of care, whereas engagement dimensions overall were not significantly associated with adverse events [52].

For nursing and healthcare managers, these findings support a work-system response rather than individual surveillance. When reduced discretionary effort, detachment, or low initiative appear within a clinical team, they should prompt examination of workload, staffing adequacy, leadership, psychological safety, and nurses’ participation in decisions affecting care. Improving nursing performance, quality of care, and patient safety, therefore, requires attention not only to outcomes but also to the conditions that enable nurses to remain engaged, available, and able to contribute fully to patient care.

At the health-system level, disengagement-related manifestations may be worth examining as potential early signals of workforce strain, particularly when they appear alongside established indicators such as turnover, absenteeism, staffing shortfalls, and workload pressure. However, given the limited and largely indirect evidence identified in this review, such indicators should be used for organizational learning and work-system improvement rather than for individual surveillance or punitive performance management.

### 4.5. QQ and Missed Nursing Care

Missed nursing care is the outcome domain where QQ’s clinical relevance is most directly testable, yet it remains the least examined: no included study tested QQ itself against missed care, care left undone, omitted care, rationed care, or implicit rationing, and the only available evidence, drawn from adjacent constructs, was limited and mixed [52,54]. These findings indicate that motivational energy may be relevant to missed care in some contexts, but they do not establish a direct QQ-missed care pathway.

This gap is clinically important because missed care captures process-level failures in nursing care delivery. Unlike distal patient outcomes, missed nursing care reflects necessary care that is omitted, delayed, rationed, or left undone under conditions such as time pressure, inadequate staffing, high workload, and insufficient resources. These omissions may involve surveillance, documentation, patient education, emotional support, care planning, discharge preparation, basic care, comfort care, or timely response to patient needs. Many depend not only on formal task completion but also on attentiveness, prioritization, initiative, communication, and sustained professional presence.

QQ is theoretically relevant to missed care because it may disrupt mechanisms that support safe practice. In constrained environments, QQ characteristics represent plausible pathways through which care delivery could be affected, although these mechanisms have not yet been directly tested.

At the same time, missed care must not be individualized or attributed simplistically to nurses’ motivation. In many cases, nurses miss care because they are forced to prioritize competing demands, not because they are unwilling to provide it. Future studies examining QQ and missed care should therefore adjust for structural and unit-level conditions to avoid nurse-blaming interpretations.

The key implication is that missed nursing care should become a priority outcome in QQ research. Longitudinal and multilevel studies are needed to determine whether QQ predicts later missed care, whether missed care and QQ emerge from shared work-system pressures, or whether any association is mediated by reduced vigor, psychological availability, team climate, or perceived organizational support. Until such evidence is available, the QQ-missed care relationship should be described as clinically important, theoretically plausible, and empirically underdeveloped.

### 4.6. Strengths and Limitations

This review has several strengths. It treats QQ as an emerging phenomenon in its own right rather than assuming interchangeability with burnout, low work engagement, or turnover intention, and its care-delivery focus is sharpened by including adjacent-construct studies only where they were empirically linked to nursing performance or patient outcomes.

Separating direct QQ evidence from adjacent evidence throughout the synthesis preserves this conceptual boundary, though it comes with a trade-off: including adjacent constructs at all introduces some conceptual heterogeneity, and the small number of direct QQ studies remains an important constraint on what the review can conclude about QQ specifically, as distinct from the broader disengagement space it sits within.

A further strength is the theory-informed synthesis. By linking work-system conditions, psychological and motivational mechanisms, disengagement-related manifestations, and care-delivery outcomes, the review offers an organizing framework for future research without claiming the proposed relationships are already empirically confirmed. The preliminary conceptual model clarifies where evidence is direct, where it is adjacent, and where the relationship between QQ and missed nursing care remains plausible but untested. As an exploratory synthesis rather than an empirically validated framework, the model should guide hypothesis generation and study design rather than be treated as a confirmed explanatory account.

These strengths should be interpreted alongside several limitations. As a scoping review, this study was designed to map the extent, nature, and characteristics of the available evidence rather than estimate pooled effects or establish causal relationships; the findings should be read as an evidence map and conceptual synthesis, not as evidence that QQ causes poorer care outcomes.

The scope of the review was also limited by language, publication type, and timeframe. Only English-language peer-reviewed journal articles published from 2022 onward were eligible; this restriction may have excluded earlier evidence on adjacent constructs, though the earlier literature was used for conceptual and contextual positioning where it did not meet the eligibility criteria for inclusion in the corpus.

Methodological limitations should also be acknowledged. No review protocol was prospectively registered or published, which may limit external auditability of methodological decisions. Methodological quality appraisal was conducted by one reviewer, although uncertain ratings were checked against the full text and MMAT guidance before final coding; the appraisal findings should therefore be interpreted as contextual information supporting evidence mapping rather than as a definitive quality ranking.

Finally, the evidence base itself is the main limitation. Most included studies were observational, relied on self-reported measures, used context-specific or non-probability samples, and employed heterogeneous operational definitions. Associations with performance and quality-of-care outcomes were drawn from five studies at most, and evidence on missed nursing care from only two. Because only two studies measured QQ directly, most care-delivery associations identified in this review come from adjacent constructs such as work engagement, vigor, and engagement–burnout profiles rather than from QQ itself. Distinguishing these constructs throughout the synthesis reduces but does not eliminate this limitation: conceptual separation cannot establish that findings for adjacent constructs generalize to QQ. The review can therefore identify a clinically important and theoretically plausible research gap, but it cannot establish whether QQ itself contributes to missed care or patient-safety outcomes.

### 4.7. Future Research Directions

Future research should move from describing QQ in nursing to testing its care-delivery relevance directly. Direct measurement is the priority because most evidence identified in this review came from adjacent constructs rather than QQ itself. Qualitative and mixed-methods studies can clarify how nurses understand, describe, and enact QQ in different clinical contexts, while intervention-oriented research can examine whether changes in work-system conditions influence disengagement-related manifestations and care-delivery processes.

Measurement refinement is central to this next phase of research. Future studies should compare existing QQ instruments, examine their dimensional structure across nursing populations and cultural contexts, and clarify whether current measures adequately capture the construct. Person-centered approaches, such as latent profile analysis and combined engagement–burnout profiling, may complement scale-based measurement by identifying configurations that total scores may obscure, particularly patterns in which nurses maintain core responsibilities while selectively reducing discretionary effort.

Longitudinal, multilevel, and multi-source designs are especially needed. Cross-sectional studies cannot determine whether adverse work-system conditions precede QQ, whether QQ predicts later changes in performance or care outcomes, or whether both emerge from shared organizational pressures. Multilevel designs would be valuable because disengagement-related manifestations may operate not only at the individual level but also within teams and units where strained conditions shape care delivery. Finally, integrating nurse-reported data with manager ratings, peer assessments, staffing indicators, adverse-event reports, patient-experience data, and routinely collected quality metrics would clarify whether QQ relates only to perceptions of care quality and safety or also to observable care-delivery processes.

## 5. Conclusions

The most notable finding of this review is not a positive association but an absence. Despite growing research on the antecedents, correlates, and workforce consequences of QQ in nursing, no included study directly tested its relationship with missed nursing care, the outcome domain through which the construct’s clinical relevance would be most concretely examined. Research attention has concentrated on why QQ occurs, leaving whether it matters for the care that patients actually receive largely untested.

This theory-informed scoping review shows that QQ in nursing has moved beyond popular discourse into early empirical investigation, but the evidence base remains narrow, conceptually heterogeneous, and only partly connected to care-delivery, patient-safety, and healthcare-quality outcomes. The review’s two-route eligibility logic revealed a clear asymmetry: nursing research has begun to define, measure, and situate QQ within adverse workplace conditions, yet its direct empirical relationship with nursing performance, patient safety, quality of care, and missed nursing care remains largely untested.

The central contribution of this review is to reframe QQ as a nursing care-delivery question. Rather than treating it as a passing workforce label, a proxy for burnout, or evidence of individual professional failure, QQ may instead signal healthcare system and organizational strain that becomes visible in how nurses calibrate their work under sustained and poorly resourced demands.

Missed care is the point at which workforce and organizational pressures may become visible as omissions, delays, and unmet patient needs, making it the outcome domain where QQ’s clinical relevance is most directly testable. Until QQ is examined directly in relation to missed nursing care, its clinical significance remains plausible but unconfirmed.

For nursing and healthcare leaders, health system managers, and policymakers, QQ should be approached as a potential work-system signal rather than an individual motivational defect. For researchers, the priority is direct measurement, longitudinal and multilevel designs, and outcome data that extend beyond self-report. Understanding whether, how, and under what conditions QQ is associated with care delivery is essential for protecting nursing workforce sustainability, nurse well-being, and safe, timely, and high-quality patient care.

## Figures and Tables

**Table 1:** PCC-based eligibility criteria.

Domain	Inclusion Criteria	Exclusion Criteria
Population	Studies involving registered nurses or licensed nursing staff providing direct patient care in clinical healthcare settings. Multidisciplinary samples were included only when nurse-specific data were reported separately or could be clearly distinguished.	Studies exclusively involving nursing students, unlicensed assistive personnel without a licensed nursing role, or non-nursing healthcare professionals without separable nursing data.
Concept	Studies directly examining QQ among nurses were eligible when they contributed evidence relevant to the review questions, including conceptualization, operationalization, measurement, level, prevalence, or associations with nursing performance, safety behavior, patient safety, quality of care, missed nursing care, care left undone, omitted care, or related care-delivery outcomes. Studies examining adjacent disengagement-related constructs were also eligible when the construct was conceptually consistent with work disengagement and empirically linked to nursing performance, safety behavior, patient safety, quality of care, missed nursing care, care left undone, or omitted care. Eligible adjacent constructs included low or diminished work engagement, reduced vigor, employee disengagement, psychological or emotional withdrawal, diminished initiative, minimal compliance, reduced discretionary effort, and burnout–engagement profiles.	Studies focusing solely on general job satisfaction, organizational culture, empowerment, leadership, affective commitment, organizational commitment, turnover intention, resilience, emotional intelligence, moral distress, workplace bullying, workplace violence, or burnout without explicit disengagement-like manifestations or without linkage to nursing performance, safety behavior, patient safety, quality of care, or missed nursing care. Studies examining work engagement only as a positive work attitude, without a clear theoretical or empirical link to disengagement-related processes and relevant care-delivery outcomes, were excluded.
Context	Clinical healthcare environments, including hospitals, critical care units, primary care settings, community care, and long-term care facilities. Studies from any geographic region or healthcare system were considered.	Non-clinical educational settings, simulation-only environments, corporate non-care contexts, or administrative roles without implications for patient care delivery.
Outcomes	Eligible outcomes included nursing work performance, task performance, contextual performance, in-role performance, extra-role performance, productivity, safety behavior, patient safety, perceived patient safety, quality of care, perceived quality of care, patient adverse events, missed nursing care, care left undone, omitted care, rationed care, or implicit rationing of nursing care. For adjacent disengagement-related constructs, empirical association with at least one of these outcomes was required.	Studies on adjacent disengagement-related constructs that did not report outcomes related to nursing performance, patient safety, quality of care, missed nursing care, care left undone, omitted care, or related care-delivery outcomes were excluded. Direct QQ studies focused exclusively on general psychosocial, organizational, or workforce correlates without contribution to the review’s care-delivery focus were not included as primary evidence.
Study Design	Empirical quantitative, qualitative, or mixed-methods studies published in peer-reviewed journals. Relevant reviews were screened for reference mining but were not included as primary evidence.	Editorials, commentaries, opinion pieces, conference abstracts without full text, dissertations, non-peer-reviewed sources, and reviews as primary evidence.
Language, Timeframe, and Accessibility	English-language articles published in peer-reviewed journals from 2022 onward and available in full text.	Non-English publications, studies published before 2022, the gray literature, and records without an accessible full text.

**Table 2:** Methodological quality appraisal of included studies using the MMAT 2018 design-specific criteria [48].

Authors	Are There Clear Research Questions? (S1)	Do the Collected Data Allow the Research Questions to be Addressed? (S2)	MMAT Q1	MMAT Q2	MMAT Q3	MMAT Q4	MMAT Q5
Mixed-methods study							
Abdullah and Bangcola [50]	Yes	Yes	Yes	Partial	Partial	Cannot tell	Partial
Quantitative non-interventional observational studies							
Wei et al. [51]	Yes	Yes	Yes	Partial	Partial	Cannot tell	Yes
Falguera et al. [52]	Yes	Yes	Yes	Partial	Partial	Cannot tell	Yes
Moisoglou et al. [53]	Yes	Yes	Yes	Partial	Yes	Cannot tell	Yes
Srulovici and Yanovich [54]	Yes	Yes	Yes	Partial	Yes	Partial	Yes
Ma et al. [55]	Yes	Yes	Yes	Partial	Yes	Cannot tell	Yes
Gillet et al. [49]	Yes	Yes	Yes	Partial	Yes	Partial	Yes

Note. S1 and S2 are the MMAT screening questions applied to all included studies: S1, Are there clear research questions? S2, Do the collected data allow the research questions to be addressed? For the mixed-methods study, Q1–Q5 correspond to the adequacy of the rationale for using a mixed-methods design (Q1), effective integration of the qualitative and quantitative components (Q2), adequate interpretation of the outputs of this integration (Q3), adequate handling of divergences and inconsistencies between qualitative and quantitative results (Q4), and adherence of each component to the quality criteria of its own methodological tradition (Q5). For the quantitative studies appraised using the MMAT quantitative descriptive criteria, Q1–Q5 correspond to the relevance of the sampling strategy (Q1), representativeness of the sample (Q2), appropriateness of the measurements (Q3), low risk of nonresponse bias (Q4), and appropriateness of the statistical analysis (Q5). Full criteria definitions follow the Mixed Methods Appraisal Tool, version 2018. These MMAT-specific questions (S1, S2, Q1–Q5) are distinct from, and should not be confused with, the review’s own four research questions (RQ1–RQ4) presented in the Introduction.

**Table 3:** Overview of included studies by design, sample, setting, and construct.

Authors	Country	Design	Sample	Setting	Construct
Abdullah and Bangcola [50]	Philippines	An exploratory mixed-methods study combining a qualitative case-study phase and a descriptive cross-sectional survey	Qualitative phase: 5 registered nurses. Quantitative phase: survey conducted among registered nurses from a target population of 214 (the final analyzable quantitative sample was not clearly reported)	Amai Pakpak Public Hospital, Marawi City, Lanao del Sur	Direct QQ
Falguera et al. [52]	Philippines	Descriptive cross-sectional study using secondary data	549 registered nurses	Government and private hospitals in Central Philippines	Work engagement
Gillet et al. [49]	Canada	Longitudinal study with measurement points 12 months apart	570 novice registered nurses at point 1 and 233 at point 2	Public healthcare system in Quebec	Work engagement and burnout profiles
Ma et al. [55]	China	Cross-sectional study with latent profile analysis	957 oncology nurses	Tertiary Grade A oncology hospitals across five provinces and municipalities	Work engagement profiles
Moisoglou et al. [53]	Greece	Cross-sectional online survey	492 clinical hospital nurses	Hospital clinical nursing settings	Quiet quitting and workplace gaslighting
Srulovici and Yanovich [54]	Israel	Nested cross-sectional study within wards	196 nurses nested in 37 wards	One public hospital (wards with overnight hospitalization)	Vigor and exhaustion
Wei et al. [51]	United States	Quantitative descriptive correlational cross-sectional survey	900 registered nurses	Multiple healthcare organizations and healthcare institutions across the United States	Work engagement

**Table 4:** Conceptualization, operationalization, and measurement of quiet quitting and disengagement-related constructs.

Authors	Conceptual Category	Conceptualization	Operationalization	Measurement	Dimensions or Indicators	Key Finding
Abdullah and Bangcola [50]	Direct quiet quitting	QQ was conceptualized as meeting job requirements without striving for overachievement and as limiting work investment while remaining employed. It was situated within adverse workplace and organizational conditions rather than being treated only as an individual attitude.	QQ was operationalized through an exploratory mixed-methods procedure in which qualitative interviews informed the subsequent quantitative survey. QQ level was then classified using a weighted scoring system.	Locally developed survey and weighted scoring approach derived from the mixed-methods process.	Indicators included poor management, disruption of work–life balance, workload and payment disparities, toxic organizational culture, risks to physical and mental health, and pressure to earn or provide.	This study provided direct conceptual evidence on QQ in nursing but used a locally developed scoring approach rather than a standardized QQ instrument.
Moisoglou et al. [53]	Direct quiet quitting	QQ was conceptualized as deliberate scaling back of discretionary effort, limitation of performance to the formal job description, detachment, reduced initiative, and reduced motivation.	QQ was operationalized as a multidimensional construct reflecting detachment, lack of initiative, and lack of motivation.	QQS, nine items, 5-point Likert scale. Higher scores indicate higher QQ.	Detachment, lack of initiative, and lack of motivation. Workplace gaslighting was measured separately through loss of self-trust and abuse of power.	This study provided direct standardized measurement evidence on QQ in nursing and allowed QQ to be distinguished from related workplace exposures.
Falguera et al. [52]	Work engagement as an inverse disengagement-related construct	Work engagement was conceptualized as a positive work-related state involving vigor, dedication, and absorption. Lower engagement indicators were treated as adjacent evidence only where empirically linked to care-delivery outcomes.	Work engagement was operationalized through global and dimensional engagement scores. Lower scores were interpreted cautiously as inverse adjacent evidence rather than as direct QQ.	Short Utrecht work engagement scale (UWES-9), 6-point Likert scale. Higher scores indicate higher work engagement.	Vigor, dedication, and absorption.	This study contributed adjacent inverse measurement evidence by operationalizing disengagement-related functioning through lower work engagement dimensions.
Gillet et al. [49]	Combined work engagement and burnout profiles	Work engagement and burnout were conceptualized as multidimensional and potentially coexisting profiles. Profiles combining low engagement with burnout-related distancing or reduced efficacy were treated as adjacent disengagement-prone configurations, not as direct QQ evidence.	Engagement and burnout were operationalized through latent profiles combining global and specific components of work engagement and burnout. Self-determination theory was used to examine psychological need satisfaction as a predictor of profile membership.	UWES-9 and Maslach Burnout Inventory. Latent profile analysis was used to identify combined profiles.	Work engagement included vigor, dedication, and absorption. Burnout included emotional exhaustion, cynicism, and reduced professional efficacy. Six profiles were identified, including profiles characterized by very low global engagement or very high global burnout.	This study showed how person-centered engagement–burnout profiles can capture disengagement-prone configurations without treating them as direct QQ evidence.
Ma et al. [55]	Work engagement profiles	Work engagement was conceptualized as a positive motivational state. A low-effort engagement profile was considered conceptually relevant to QQ because it reflected reduced work investment, but it was not treated as equivalent to QQ.	Work engagement was operationalized through person-centered engagement profiles derived from responses to the questionnaire.	Chinese version of the UWES-9, 7-point Likert scale, followed by latent profile analysis.	Latent profiles were derived from response patterns across the nine work engagement items representing vitality, dedication, and focus. Three profiles were retained: low effort and coping type, moderately balanced type, and highly efficient and focused type.	This study contributed adjacent person-centered evidence by identifying a low-effort engagement profile conceptually close to, but distinct from QQ.
Srulovici and Yanovich [54]	Vigor and exhaustion within a JD-R pathway	Nurse vigor was conceptualized as motivational energy and resilience in dealing with everyday work difficulties, whereas exhaustion reflected energetic depletion under workload demands. Reduced vigor was treated as an adjacent disengagement-related indicator, while exhaustion was retained as related contextual evidence rather than as equivalent to QQ.	Vigor and exhaustion were operationalized as distinct motivational and energetic pathways within a JD-R framework.	Vigor was measured with the 5-item vigor subscale of the work engagement scale. Exhaustion was measured with the 5-item exhaustion subscale of the Maslach Burnout Inventory.	Nurse vigor reflected motivational energy and resilience at work. Nurse exhaustion reflected energetic depletion.	This study distinguished reduced motivational energy from energetic depletion, supporting a more precise interpretation of adjacent constructs.
Wei et al. [51]	Work engagement as an inverse disengagement-related construct	Work engagement was conceptualized as a positive work-related state involving vigor, dedication, and absorption. Lower engagement was interpreted cautiously as inverse adjacent evidence rather than as direct QQ.	Work engagement was operationalized through total and dimensional engagement scores. Lower work engagement was used as adjacent inverse evidence only in relation to perceived quality of care.	Utrecht work engagement scale-17 (UWES 17), 7-point scale. Higher scores indicate higher work engagement.	Vigor, dedication, and absorption.	This study contributed adjacent inverse measurement evidence by operationalizing disengagement-related functioning through lower total and dimensional work engagement scores.

**Table 5:** Study-level findings on performance and care-related outcomes.

Authors	Outcome Examined	Key Finding
Falguera et al. [52]	Perceived quality of care and patient adverse events	Dedication was the only work engagement dimension associated with perceived quality of care. No significant associations were reported between work engagement dimensions and patient adverse events.
Gillet et al. [49]	In-role performance and extra-role performance	Profiles characterized by low dedication, reduced efficacy, and high cynicism were associated with poorer in-role and extra-role performance.
Ma et al. [55]	Nurse safety behavior	The low-effort engagement profile was associated with the lowest nurse safety behavior scores.
Moisoglou et al. [53]	Perceived quality of care and perceived patient safety	Workplace gaslighting was associated with higher QQ and with poorer perceived quality-of-care and patient-safety ratings; QQ itself was not tested as a predictor of these outcomes.
Wei et al. [51]	Perceived quality of care	Nurses reporting lower work engagement were less likely to report high perceived quality of care.

**Table 6:** Study-level measures and findings on Missed Nursing Care and Care Left Undone.

Authors	Outcome Examined	Instrument	Key Finding
Falguera et al. [52]	Missed nursing care and care left undone	13-item missed nursing care [21]	Work engagement dimensions were not significantly associated with missed nursing care in this sample.
Srulovici and Yanovich [54]	Missed nursing care	22-item MISSCARE survey [20]	Lower vigor, rather than exhaustion, was associated with more frequent missed nursing care.

**Table 7:** Conceptual distinctions between quiet quitting and adjacent disengagement-related constructs.

Construct	Core Conceptualization	Role Continuity	Primary Distinguishing Feature	Typical Measurement Approach
Quiet quitting	Deliberate restriction of discretionary effort while formal duties continue to be met [11,16]	Nurse remains in role	Intentional and selective narrowing of effort within continued employment	Quiet Quitting Scale [9]
Burnout	Emotional exhaustion, depersonalization, and reduced personal accomplishment [17]	Nurse may remain in role	Reflects energetic depletion rather than a deliberate decision to restrict effort	Maslach Burnout Inventory [49,54]
Work engagement (low)	Reduced vigor, dedication, and absorption [17]	Nurse remains in role	Reflects diminished energy and involvement rather than intentional withdrawal of effort	Utrecht Work Engagement Scale [49,51,52,55]
Turnover intention	Intention or desire to leave the job [15,18,19]	Nurse remains employed at measurement, but the construct is exit-oriented	Concerns about intention to leave rather than continued reduced investment within the role	Direct Self-Report Items on Intention to Leave [15,18,19]

## Data Availability

No new primary data were generated in this study. All data charted and synthesized in this review are available in the included articles and the Supplementary Materials. Data sharing is not applicable to this article.

## Supplementary Materials

The following supporting information can be downloaded at: https://www.mdpi.com/article/10.3390/healthcare14142145/s1, Table S1: Preferred Reporting Items for Systematic reviews and Meta-Analyses extension for Scoping Reviews (PRISMA-ScR) Checklist [45]; Table S2: Search Strategy and Results by Database.

## Abbreviations

The following abbreviations are used in this manuscript:

QQ	Quiet Quitting
PCC	Population–Concept–Context
PRISMA-ScR	Preferred Reporting Items for Systematic Reviews and Meta-Analyses Extension for Scoping Reviews
MMAT	Mixed Methods Appraisal Tool
JD-R	Job Demands–Resources Model
