# Supplementary material for: Quiet Quitting and Disengagement-Related Constructs in Nursing: A Theory-Informed Scoping Review
Source: Healthcare (Basel). 2026 Jul 16;14(14):2145. doi: 10.3390/healthcare14142145 (PMC13411971; doi:10.3390/healthcare14142145)
Supplement: Supplementary file 1 [file healthcare-14-02145-s001.zip › Table S2. research strategy queries.pdf]

Table S2. Search Strategy and Results by Database

Database	Search Strategy	Results (n)
**PubMed**	("Quiet Quitting"[Title/Abstract] OR "quiet quit*"[Title/Abstract] OR "work disengagement"[Title/Abstract] OR "employee disengagement"[Title/Abstract] OR "job disengagement"[Title/Abstract] OR "psychological withdrawal"[Title/Abstract] OR "work withdrawal"[Title/Abstract] OR "employee withdrawal"[Title/Abstract] OR "reduced work engagement"[Title/Abstract] OR "low work engagement"[Title/Abstract] OR "reduced vigour"[Title/Abstract] OR "reduced vigor"[Title/Abstract] OR "minimal compliance"[Title/Abstract] OR "reduced discretionary effort"[Title/Abstract] OR "burnout engagement profile*"[Title/Abstract] OR "burnout-engagement profile*"[Title/Abstract] OR "work engagement"[Title/Abstract] OR "Burnout, Psychological"[MeSH Terms])<br>AND ("Nurses"[MeSH Terms] OR "Nursing Staff"[MeSH Terms] OR nurse*[Title/Abstract] OR "registered nurse*"[Title/Abstract] OR "nursing staff"[Title/Abstract] OR "clinical nurse*"[Title/Abstract])<br>AND ("Patient Safety"[MeSH Terms] OR "Quality of Health Care"[MeSH Terms] OR "Nursing Care"[MeSH Terms] OR "patient safety"[Title/Abstract] OR "safety behaviour"[Title/Abstract] OR "safety behavior"[Title/Abstract] OR "nurse safety behaviour"[Title/Abstract] OR "nurse safety behavior"[Title/Abstract] OR "quality of care"[Title/Abstract] OR "care quality"[Title/Abstract] OR "perceived quality of care"[Title/Abstract] OR "work performance"[Title/Abstract] OR "job performance"[Title/Abstract] OR "task performance"[Title/Abstract] OR "contextual performance"[Title/Abstract] OR "in-role performance"[Title/Abstract] OR "extra-role performance"[Title/Abstract] OR "patient adverse event*"[Title/Abstract] OR "adverse event*"[Title/Abstract] OR "missed nursing care"[Title/Abstract] OR "missed care"[Title/Abstract] OR "care left undone"[Title/Abstract] OR "omitted care"[Title/Abstract] OR "rationed care"[Title/Abstract] OR "implicit rationing"[Title/Abstract])<br>AND ("2022/01/01"[Date - Publication] : "3000"[Date - Publication]) AND english[Language]<br>*Filters applied: Full text, English, Humans, MEDLINE, 2022/1/1–2026/3/19*	**1,754**
**Scopus**	TITLE-ABS-KEY ("quiet quitting" OR "quiet quit*" OR "work disengagement" OR "employee disengagement" OR "job disengagement" OR "psychological withdrawal" OR "work withdrawal" OR "employee withdrawal" OR "reduced work engagement" OR "low work engagement" OR "reduced vigour" OR "reduced vigor" OR "minimal compliance" OR "reduced discretionary effort" OR "burnout engagement profile*" OR "burnout-engagement profile*" OR "work engagement")<br>AND TITLE-ABS-KEY (nurse* OR "registered nurse*" OR "nursing staff" OR "clinical nurse*")<br>AND TITLE-ABS-KEY ("patient safety" OR "safety behaviour" OR "safety behavior" OR "nurse safety behaviour" OR "nurse safety behavior" OR "quality of care" OR "care quality" OR "perceived quality of care" OR "work performance" OR "job performance" OR "task performance" OR "contextual performance" OR "in-role performance" OR "extra-role performance" OR "patient adverse event*" OR "adverse event*" OR "missed nursing care" OR "missed care" OR "care left undone" OR "omitted care" OR "rationed care" OR "implicit rationing")<br>AND PUBYEAR > 2021 AND LANGUAGE (english) AND DOCTYPE (ar) AND (LIMIT-TO (DOCTYPE, "ar")) AND (LIMIT-TO (LANGUAGE, "English"))	**144**
**Web of Science Core Collection**	TS=("quiet quitting" OR "quiet quit*" OR "work disengagement" OR "employee disengagement" OR "job disengagement" OR "psychological withdrawal" OR "work withdrawal" OR "employee withdrawal" OR "reduced work engagement" OR "low work engagement" OR "reduced vigour" OR "reduced vigor" OR "minimal compliance" OR "reduced discretionary effort" OR "burnout engagement profile*" OR "burnout-engagement profile*" OR "work engagement")<br>AND TS=(nurse* OR "registered nurse*" OR "nursing staff" OR "clinical nurse*")<br>AND TS=("patient safety" OR "safety behaviour" OR "safety behavior" OR "nurse safety behaviour" OR "nurse safety behavior" OR "quality of care" OR "care quality" OR "perceived quality of care" OR "work performance" OR "job performance" OR "task performance" OR "contextual performance" OR "in-role performance" OR "extra-role performance" OR "patient adverse event*" OR "adverse event*" OR "missed nursing care" OR "missed care" OR "care left undone" OR "omitted care" OR "rationed care" OR "implicit rationing")	**130**

Database	Search Strategy	Results (n)
**Cochrane Library**	("quiet quitting" OR "quiet quit*" OR "work disengagement" OR "employee disengagement" OR "job disengagement" OR "psychological withdrawal" OR "work withdrawal" OR "reduced work engagement" OR "low work engagement" OR "work engagement" OR "reduced vigour" OR "reduced vigor" OR "burnout engagement profile*" OR "burnout-engagement profile*")<br>AND (nurse* OR "registered nurse*" OR "nursing staff" OR "clinical nurse*")<br>AND ("patient safety" OR "quality of care" OR "care quality" OR "work performance" OR "job performance" OR "safety behaviour" OR "safety behavior" OR "missed nursing care" OR "missed care" OR "care left undone" OR "omitted care" OR "rationed care")<br>*Publication years: 2022–2026. Language: English. Trials, reviews, and CENTRAL records screened for potentially relevant empirical studies.*	**1**
**CINAHL (via EBSCOhost)**	**Search A:**<br>(TI ("quiet quitting" OR "quiet quit*") OR AB ("quiet quitting" OR "quiet quit*")) AND (TI (nurse* OR "registered nurse*" OR "nursing staff" OR "clinical nurse*" OR "hospital nurse*") OR AB (nurse* OR "registered nurse*" OR "nursing staff" OR "clinical nurse*" OR "hospital nurse*") OR MH "Nurses+" OR MH "Registered Nurses+" OR MH "Nursing Staff+")<br>**Search B:**<br>(TI ("work disengagement" OR "employee disengagement" OR "job disengagement" OR "psychological withdrawal" OR "work withdrawal" OR "employee withdrawal" OR "reduced work engagement" OR "low work engagement" OR "reduced engagement" OR "work engagement" OR "reduced vigour" OR "reduced vigor" OR "low vigour" OR "low vigor" OR "minimal compliance" OR "reduced discretionary effort" OR "lack of initiative" OR "lack of motivation" OR "detachment" OR "burnout engagement profile*" OR "burnout-engagement profile*" OR "engagement burnout profile*" OR "engagement-burnout profile*")<br>OR AB ("work disengagement" OR "employee disengagement" OR "job disengagement" OR "psychological withdrawal" OR "work withdrawal" OR "employee withdrawal" OR "reduced work engagement" OR "low work engagement" OR "reduced engagement" OR "work engagement" OR "reduced vigour" OR "reduced vigor" OR "low vigour" OR "low vigor" OR "minimal compliance" OR "reduced discretionary effort" OR "lack of initiative" OR "lack of motivation" OR "detachment" OR "burnout engagement profile*" OR "burnout-engagement profile*" OR "engagement burnout profile*" OR "engagement-burnout profile*")<br>OR MH "Burnout, Professional+")<br>AND (TI (nurse* OR "registered nurse*" OR "nursing staff" OR "clinical nurse*" OR "hospital nurse*") OR AB (nurse* OR "registered nurse*" OR "nursing staff" OR "clinical nurse*" OR "hospital nurse*") OR MH "Nurses+" OR MH "Registered Nurses+" OR MH "Nursing Staff+")<br>AND (TI ("patient safety" OR "safety behaviour" OR "safety behavior" OR "nurse safety behaviour" OR "nurse safety behavior" OR "quality of care" OR "care quality" OR "perceived quality of care" OR "nursing care quality" OR "work performance" OR "job performance" OR "task performance" OR "contextual performance" OR "in-role performance" OR "extra-role performance" OR "patient adverse event*" OR "adverse event*" OR "missed nursing care" OR "missed care" OR "care left undone" OR "omitted care" OR "rationed care" OR "rationed nursing care" OR "implicit rationing")<br>OR AB ("patient safety" OR "safety behaviour" OR "safety behavior" OR "nurse safety behaviour" OR "nurse safety behavior" OR "quality of care" OR "care quality" OR "perceived quality of care" OR "nursing care quality" OR "work performance" OR "job performance" OR "task performance" OR "contextual performance" OR "in-role performance" OR "extra-role performance" OR "patient adverse event*" OR "adverse event*" OR "missed nursing care" OR "missed care" OR "care left undone" OR "omitted care" OR "rationed care" OR "rationed nursing care" OR "implicit rationing")<br>OR MH "Patient Safety+" OR MH "Quality of Health Care+" OR MH "Nursing Care+")	**883**
**Google Scholar (supplementary search)**	**Search A:**<br>"quiet quitting" nurses "quality of care" OR "patient safety" OR nurses performance OR "missed nursing care"<br>**Search B:**<br>"work engagement" nurses "quality of care" OR "patient safety" OR nurses performance OR "missed nursing care"	**349**
